# Tropical wetlands: A missing link in the global carbon cycle?

**DOI:** 10.1002/2014GB004844

**Published:** 2014-12-04

**Authors:** Sofie Sjögersten, Colin R Black, Stephanie Evers, Jorge Hoyos-Santillan, Emma L Wright, Benjamin L Turner

**Affiliations:** 1 School of Biosciences, University of NottinghamNottingham, UK; 2 School of Biosciences, University of NottinghamKuala Lumpur, Malaysia; 3 Smithsonian Tropical Research InstitutePanama City, Panama

**Keywords:** carbon dioxide, decomposition, methane, net primary productivity, tropical, wetland

## Abstract

Tropical wetlands are not included in Earth system models, despite being an important source of methane (CH_4_) and contributing a large fraction of carbon dioxide (CO_2_) emissions from land use, land use change, and forestry in the tropics. This review identifies a remarkable lack of data on the carbon balance and gas fluxes from undisturbed tropical wetlands, which limits the ability of global change models to make accurate predictions about future climate. We show that the available data on in situ carbon gas fluxes in undisturbed forested tropical wetlands indicate marked spatial and temporal variability in CO_2_ and CH_4_ emissions, with exceptionally large fluxes in Southeast Asia and the Neotropics. By upscaling short-term measurements, we calculate that approximately 90 ± 77 Tg CH_4_ year^−1^ and 4540 ± 1480 Tg CO_2_ year^−1^ are released from tropical wetlands globally. CH_4_ fluxes are greater from mineral than organic soils, whereas CO_2_ fluxes do not differ between soil types. The high CO_2_ and CH_4_ emissions are mirrored by high rates of net primary productivity and litter decay. Net ecosystem productivity was estimated to be greater in peat-forming wetlands than on mineral soils, but the available data are insufficient to construct reliable carbon balances or estimate gas fluxes at regional scales. We conclude that there is an urgent need for systematic data on carbon dynamics in tropical wetlands to provide a robust understanding of how they differ from well-studied northern wetlands and allow incorporation of tropical wetlands into global climate change models.

## 1. Introduction

Tropical wetlands play an important role in the global carbon (C) cycle [*Page et al.*, 2011]. Currently, they are under considerable pressure from agriculture [*Houghton*, 2012] resulting in substantially increased carbon dioxide (CO_2_) emissions from these ecosystems. For example, 1–3% of annual fossil fuel emissions or 355–855 Mt C year^−1^ in Indonesia alone [*Hooijer et al.*, 2010] are estimated to originate from tropical peatlands. Undisturbed tropical wetlands emit between 85 and 184 Tg of methane (CH_4_) each year, accounting for two thirds of global emissions from wetlands [e.g., *Richey et al.*, 2002; *Jauhiainen et al.*, 2005; *Hooijer et al.*, 2006; *Nahlik and Mitsch*, 2011; *Melton et al.*, 2013].

The dominant wetland ecosystems in the tropics are forested peatlands, swamps, and floodplains (Table1) [*Aselmann and Crutzen*, 1989]. Of these, only peatlands accumulate substantial C deposits (between 0.5 and 11 m deep) [*Phillips et al.*, 1997; *Page et al.*, 1999; *Shimada et al.*, 2001; *Hope et al.*, 2005; *Page et al.*, 2011; *Lähteenoja et al.*, 2012]. However, controls on the formation of deep peats in the tropics are not well understood. As expected from their capacity for C accumulation, tropical peatlands comprise a significant proportion of terrestrial C: an estimated 89 Gt C or 19% of the C stored in peatlands worldwide [*Page et al.*, 2011]. Accumulation of C in tropical peatlands is under threat from land use and climate change, which can transform tropical wetlands into C sources [*Furukawa et al.*, 2005; *Laiho*, 2006; *Meehl et al.*, 2007; *Hooijer et al.*, 2010].

**Table 1:** Description of Wetland Types^a^

Wetland Type	Description	Area (km^2^)
Swamps	Forested freshwater wetlands on waterlogged or inundated soils where little or no peat accumulation takes place. For this review we have limited data to forested system.	230,000
Peatlands	Peat producing wetlands in moist climates where organic materials have accumulated over long periods.	441,000
Floodplains	Periodically follower areas along rivers or lakes showing considerable variation in vegetation cover. In the Amazon flood plain two separate systems are defined *Varzea forests* which are feb by muddy rivers and *Igapo forests* located in blackwater and clearwater tributaries	715,000

a For this review we have limited data to forested systems.

**Figure 1:**
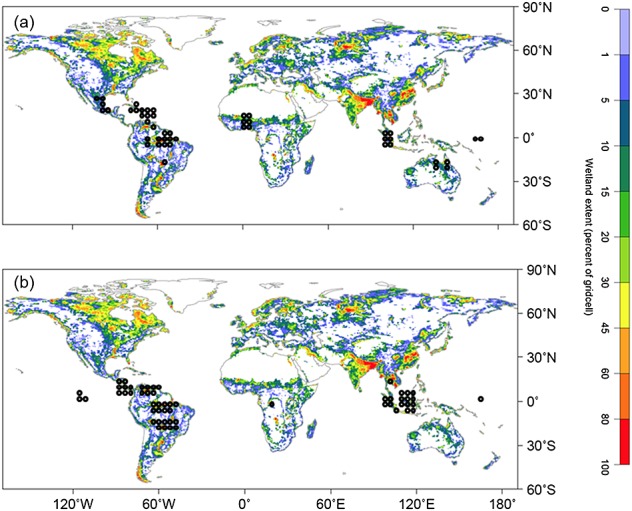
The wetland map is based on remotely sensed inundation data and GIEMS refers to the Global Inundation Extent from Multi-Satellites; the GIEMS inundation data set is plotted as the mean annual maximum value across between 1993 and 2004 [*Melton et al.*, 2013]. (a) The spatial distribution of NPP data sets (data in Table2) and (b) greenhouse gas flux data sets (data in Table4).

There are considerable uncertainties regarding the spatial extent of tropical wetlands (Figure1). Observational data suggest that tropical wetland areas range between 2.8 and 6.0 × 10^6^ km^2^, while models predict a much larger range (1.3–38.8 × 10^6^ km^2^) [*Melton et al.*, 2013]. Uncertainties regarding the relative distribution of tropical wetland types are even larger; areal estimates of different wetland types are presented in Table1 [*Aselmann and Crutzen*, 1989; *Page et al.*, 2011]. Given the contrasting environmental conditions associated with these different wetland types (e.g., peat accumulation and nutrient-poor conditions in peatlands and seasonal variation in the degree of inundation in floodplain systems), tropical wetlands are not only expected to differ in C accumulation as peat but also their release of CO_2_ and CH_4_.

**Table 2:** Net Primary Productivity Based on Litterfall Data in a Range of Forested Tropical Wetlands

Region, Country	Forest Type, Site Name	Soil Type	NPP_total_^a^ (g C m^−2^ yr^−1^)	Reference
Puerto Rico	*Pterocarpus officinalis* forest	Organic	1277	*Easse and Aide* [1999]
Luquillo, Puerto Rico	Flood plain palm forest	Organic	616	*Frangi and Lugo* [1998b]
Puerto Rico	*Prestoea montana* forest	Organic	1929	*Frangi and Lugo* [1985]
Veracruz, Mexico	Forested wetlands, Apompal	Organic	1056	*Mata et al.* [2012]
Veracruz, Mexico	Forested wetlands, Mancha	Organic	1101	*Mata et al.* [2012]
Veracruz, Mexico	Forested wetlands, Chica	Organic	1691	*Mata et al.* [2012]
Veracruz, Mexico	Forested wetlands, Cienaga	Mineral	1566	*Mata et al.* [2012]
Veracruz, Mexico	Forested wetlands, Salado	Organic	1419	*Mata et al.* [2012]
Puerto Rico	*Pterocarpus officinalis* forest, Mayaguez	Organic	1600	*Alvarez-Lopez* [1990]
Puerto Rico	*Pterocarpus officinalis* forest, Patillas	Organic	1351	*Alvarez-Lopez* [1990]
Puerto Rico	*Pterocarpus officinalis* forest, Dorado	Mineral	987	*Alvarez-Lopez* [1990]
Guadeloupe	*Pterocarpus officinalis* swamp forest	Organic	1476	*Miegot and Imbert* [2012]
Guadeloupe	*Pterocarpus officinalis* swamp forest	Organic	1606	*Miegot and Imbert* [2012]
Guadeloupe	*Pterocarpus officinalis* swamp forest	Organic	1189	*Miegot and Imbert* [2012]
Panama	Riverine forest	Mineral	1318	*Golley et al.* [1975]
Peru	Flood plain forest, high restinga	Mineral	796	*Nebel et al.* [2001]
Peru	Flood plain forest, low restinga	Mineral	810	*Nebel et al.* [2001]
Peru	Flood plain forest, *Tahuampa*	Mineral	787	*Nebel et al.* [2001]
Orinoco Llanos, Venezuela	Palm swamp forest, flood-prone	Organic	560	*San-José et al.* [2010]
Orinoco Llanos, Venezuela	Palm swamp forest, flood plain	Organic	2438	*San-José et al.* [2010]
Brazil	Swamp forest	Mineral	647	*Terror et al.* [2011]
Pantanal, Brazil	Flooded forest	Mineral	1021	*Haase* [1999]
Manaus, Brazil	Swamp forest, Igapo	Organic	772	*Adis et al.* [1979]
Manaus, Brazil	Flood plain forest	Mineral	726	*Franken et al.* [1979]
Manaus, Brazil	Swamp forest	Organic	760	*Franken et al.* [1979]
Para, Brazil	Swamp forest	Organic	976	*Klinge* [1978]
Para, Brazil	Flood plain forest	Mineral	193	*Klinge* [1978]
Para, Brazil	Swamp forest	Organic	874	*Silva and Lobo* [1982]
Para, Brazil	Flood plain forest	Mineral	976	*Silva and Lobo* [1982]
Para, Brazil	Flood plain forest	Mineral	1566	*Cattanio et al.* [2004]
Amazonia	Floodplain forest, varzea, 40 year old	Mineral	1190	*Naiman* [2005]
Amazonia	Floodplain forest, varzea, 80 year old	Mineral	1680	*Naiman* [2005]
Australia	Flood plain forest *Mimosa pigra*	Mineral	430	*Payntner* [2005]
Australia	Flood plain forest, *Melaleuca* spp.—Mangrove, northeastern Queensland	Mineral	470	*Duke* [1982]
Australia	*Melaleuca* spp. forest, Magela flood plain	Mineral	350	*Finlayson et al.* [1993]
Australia	*Melaleuca* spp forest, Magela flood plain	Mineral	750	*Finlayson* [1988]
Ivory coast	Water logged forest, VG	Mineral	919	*Devineau* [1976]
Ivory coast	Riverine forest, TR6	Mineral	783	*Devineau* [1976]
Ivory coast	Riverine forest, gallery, MS	Mineral	965	*Devineau* [1976]
Ivory coast	Riverine forest, gallery, TR4	Mineral	704	*Devineau* [1976]
Ivory coast	Riverine forest, gallery, BD	Mineral	874	*Devineau* [1976]
Ivory coast	Riverine forest, gallery, TR2	Mineral	602	*Devineau* [1976]
Malaysia, Tasek Bera	Riverine forest, *Eugenia* swamp	Organic	1039	*Furtado et al.* [1980]
Sumatra, Indonesia	Peat swamp forest, PS3	Organic	1351	*Brady* [1997]
Sumatra, Indonesia	Peat swamp forest, SE6	Organic	829	*Brady* [1997]
Sumatra, Indonesia	Peat swamp forest, PI6	Organic	783	*Brady* [1997]
Sumatra, Indonesia	Peat swamp forest, PI9	Organic	624	*Brady* [1997]
Sumatra, Indonesia	Peat swamp forest, PI12	Organic	624	*Brady* [1997]
Yela, Micronesia	Peat swamp forest	Organic	1689	*Chimner and Ewel* [2005]
Yewak, Micronesia	Peat swamp forest	Organic	1716	*Chimner and Ewel* [2005]

a NPP_total_ is based on conversion of NPP_canopy_ using NPP_total_ = 2.27^*^NPP_canopy_ [*Malhi et al.*, 2011], where total NPP was not reported.

b Data from 1980.

The rate of increase in CH_4_ concentration in the atmosphere has varied during the past three decades reassuming its increase after 2006 to *ca*. 6 Tg CH_4_ y^−1^ [*Kirschke et al.*, 2013]; with tropical wetlands playing a major role in the renewed increase of atmospheric CH_4_ [*IPCC*, 2013]. The magnitude of this increase has been observed to differ depending on whether the estimate is based on a top-down (atmospheric inversion models) or a bottom-up (process-based models; adding up independently estimated flux components) analytical approach. Higher estimates have been reported using the bottom-up approach, where the estimates of fluxes from natural wetlands carry an uncertainty of at least 50% [*Kirschke et al.*, 2013]. The uncertainty in the bottom-up approach of the CH_4_ emissions from wetlands is mainly due to the lack of a reliable estimate of the global extent of wetlands [*Melton et al.*, 2013] and to the scarcity of wetland CH_4_ flux measurements [*Riley et al.*, 2011].

Our rudimentary understanding of CH_4_ emissions in the tropics is underlined by the discrepancy between emissions of CH_4_ from the surface of wetlands and the high concentrations of this gas in the tropical atmosphere [*Melack et al.*, 2004; *Miller et al.*, 2007; *Bergamaschi et al*., 2009; *Bloom et al.*, 2010]; with CH_4_ emissions from top-down and bottom-up approaches differing the most in tropical South America [*Kirschke et al.*, 2013]. Addressing this knowledge gap is of particular importance as models predict a global increase in CH_4_ emissions of 77%, due largely to increased emissions from existing tropical wetlands in response to increasing temperatures [*Shindell et al.*, 2004]. The model used by *Shindell et al.* [2004] calculates CH_4_ emissions based on relationships between temperature, water table depth, and net primary productivity (NPP). Some progress has been made in testing these relationships [*Walter and Heimann*, 2000], but data are limited, particularly regarding NPP and temperature responses; such issues must be considered in greater detail [*Farmer et al.*, 2012].

Several existing wetland modeling tools may be suitable for application to tropical peatlands and some might be useful in Earth system models [*Farmer et al.*, 2012]. However, the inclusion of tropical wetlands in such models is hampered by a lack of suitable data to validate them. Current models of global CH_4_ emissions [*Bridgham et al.*, 2013; *Melton et al.*, 2013] use estimates of tropical CH_4_ fluxes from a small number of review papers [e.g., *Matthews and Fung*, 1987; *Aselmann and Crutzen*, 1989; *Bartlett and Harriss*, 1993] that estimated CH_4_ emissions from a limited number of measurements. It is therefore not surprising that outputs from wetland models that estimate current CH_4_ emissions from tropical areas vary widely, with values between 85 ± 7 and 184 ± 11 Tg CH_4_ year^−1^ [*Melton et al.*, 2013]. Without appropriate data on C dynamics from undisturbed tropical wetlands, it will be difficult to predict how degradation of these systems will impact on global climate. Key input data needed to model C dynamics in tropical wetlands are aboveground and belowground net primary productivity (NPP), litter input and decay, and information on soil properties, including nutrient status, and hydrology [*Farmer et al.*, 2012]. Good quality CO_2_ and CH_4_ flux data, i.e., data accounting for temporal and spatial variability in fluxes are also needed to evaluate model predictions and close the gap between top down and bottom up modeling approaches [*Farmer et al.*, 2012].

Compared to the more intensively studied boreal and temperate peatlands, tropical peatlands are poorly understood with respect to the controls on decomposition and C storage; the C sink strength of tropical peatlands therefore remains poorly quantified [*Dommain et al.*, 2011]. However, tropical wetlands have common characteristics, such as high mean annual temperature with little seasonal variation, high rainfall, generally high hydraulic conductivity at the surface in the case of peatlands, and the presence of overstorey rainforest providing the main input of organic matter [*Page et al.*, 1999; *Sjögersten et al.*, 2010; *Lähteenoja and Page*, 2011; *Wright et al.*, 2011]. Carbon accumulation in ecosystems is determined by the balance between inputs and output. In high-latitude wetlands, the main control of C accumulation is slow decomposition of recalcitrant litter inputs, often *Sphagnum* spp., in cold wet soils [*Clymo*, 1984], whereas the situation in the tropics is less well understood. In contrast to cold regions, temperature is unlikely to be a major factor in limiting decomposition. The recalcitrance of litter inputs is less constrained as they are produced from different plant tissue types and plant species. *Chimner and Ewel* [2005] suggested that relatively slow root decomposition may be instrumental in the formation of tropical peat, implying that root production rate is important in determining C balance. However, the relationship between NPP and long-term C storage within tropical wetlands has not been explored.

We calculated current C balances for a wide range of tropical wetlands by compiling data for long-term net C accumulation rates and CO_2_ and CH_4_ emissions from flooded tropical wetlands/peatlands. It was anticipated that C accumulation rates would be greater in tropical than in temperate and boreal peatlands, but that CO_2_ and CH_4_ emissions would be high due to the substantial inputs of fresh litter and stable high temperatures. The hypothesis that C accumulation in tropical peatlands is driven by slow decomposition rather than high NPP was tested by comparing decomposition rates and NPP with tropical wetlands that do not accumulate peat.

## 2. Methods

### 2.1. Data Collation

The Web of Knowledge and Google Scholar were used to collate information on CO_2_ and CH_4_ fluxes, peat depth, NPP, and C accumulation from the relevant published literature using the following search terms: Tropical, Amazon, Pantanal, Africa, Southeast Asia, peatlands, wetlands, methane, peat, carbon dioxide, biomass, litter, NPP, and root. Based on the references obtained, all relevant original research pertaining to forested tropical wetland areas was used to identify additional references. We consider only freshwater wetlands.

To assess litter decomposition rates, a data set of decay constants (*k*) was compiled for different litter types from in situ decomposition in tropical and subtropical wetlands, with high *k* values corresponding to more rapid decay. Half times (half time = ln(2)/*k*) were calculated for different tissue types.

### 2.2. Data Processing and Analysis

We used two approaches to estimate NPP, (i) by summing C inputs and (ii) by using a conversion between litter production and total NPP. To construct a C balance for wetlands on organic and mineral soil, using the first approach, plant production was estimated by summing leaf litterfall, reproductive litterfall (flowers, fruit, and seed), branch litterfall, other litter (e.g., chaff), wood increment, and fine root production. No data were found for coarse woody debris or coarse root production. Published data for litter production were generally presented as mass of material, for conversion to C inputs a 50% C content was assumed [*Wright et al.*, 2013]. We assumed that data for some of the litter pools needed for estimating NPP this way would be limited. Therefore, we used our second approach for estimating NPP. This was based on a linear relationship between NPP_total_ and NPP_canopy_ reported for lowland rainforest [*Malhi et al.*, 2011], and we chose this approach since data availability for canopy litter production in tropical wetlands was the most regularly measured component of the C inputs. The relationship was used to estimate NPP based on the assumption that NPP_total_ = 2.27(NPP_canopy_). NPP_canopy_ was calculated as leaf litter + reproductive litterfall + branch litterfall + other litter again assuming a C content of 50% to convert litterfall to C inputs. Net ecosystem production (NEP) was calculated by subtracting total C losses (in the form of average gaseous losses as CO_2_ and CH_4_ and aquatic losses as dissolved organic carbon (DOC) across all sites from which data were available) from the substrate from NPP_total_.

Calculations of NEP were separated between the organic and mineral soil components, and estimates of heterotrophic respiration were based on upscaling of short-term in situ ground surface flux measurements to the annual scale to enable comparison with litter inputs. The measurements of surface CO_2_ flux combine both autotrophic and heterotrophic respiration; as measurements were largely collected during the daytime period, this may have introduced bias within the data. Furthermore, collection of flux data during different seasons may also have influenced the balance between C inputs and output (inputs were based on litterfall data normally collected over an annual cycle). Potential data limitations are highlighted in the discussion.

Tests for significant differences in CO_2_ and CH_4_ fluxes and NPP_total_ between tropical wetland types (e.g., peat forming versus wetlands on mineral soil) and geographical regions were conducted using an unbalanced analysis of variance (ANOVA). CO_2_ and CH_4_ flux data were square root and log transformed, respectively, to meet the normality assumption of ANOVA. All statistical analysis was carried out using GENSTAT version 15. To assess the impacts of data gaps in the C balance, we carried out a sensitivity analysis calculating potential errors associated with particular data gaps relative to the total C inputs using existing studies from either tropical wetlands or tropical lowland rainforest system.

## 3. Carbon Accumulation

Carbon accumulates in both mineral and peat-forming tropical wetlands and a wide range of peat accumulation rates have been reported for tropical peatlands; for example, *Chimner and Ewel* [2005] estimated accumulation on the island of Kosrae in Micronesia to be 300 g C m^−2^ yr^−1^, at the higher end of the range reported for the tropics. In Kalimantan, mean accumulation rates were estimated to be 31–77 g C m^−2^ yr^−1^ [*Dommain et al.*, 2011] and 94 g C m^−2^ yr^−1^ [*Moore et al.*, 2013], while comparable values of 39–85 g C m^−2^ yr^−1^ have been reported for Peruvian Amazon peatlands [*Lähteenoja et al.*, 2009] and 43–55 g C m^−2^ yr^−1^ in Panamanian peatlands (J. Hoyos, unpublished data, 2014). Furthermore, peat accumulation rates appear to be greater in coastal lowland peatlands than in inland peatlands [*Dommain et al.*, 2011]. *Hirano et al.* [2009] reported that net ecosystem C production (NEP) in a drained peatland forest in Kalimantan ranged from 296 to 594 g C m^−2^ yr^−1^, at the upper end of range of long-term C accumulation rates.

Carbon accumulation is also substantial in depositional sedimentary flood plain systems. *Moreira-Turcq et al.* [2004] suggested a rate of 100 g C m^−2^ yr^−1^ for the varzea of the Amazon, while *Devol et al.* [1984] suggested a rate of 44 g C m^−2^ yr^−1^ based on depositional systems connected to the Amazon for only 6 months of the year. In Lake Rawa Danau, West Java, Indonesia, sedimentary deposition of organic C was lower at 11.75 g C m^−2^ yr^−1^. Flux data are lacking for C inputs into the Bengal delta plain, even though this region may represent an important store given the high outflow of sediments with C contents ranging between 0.05 and 1.4% [*Datta et al.*, 1999].

Carbon accumulation rates in boreal and temperate peatlands are generally lower than in the tropics, although substantial variation occurs depending on peatland type, with values as high as 132–198 g C m^−2^ yr^−1^ being recorded for bogs in the USA [*Craft et al.*, 2008]. However, lower peat accretion rates are also common; for example, rates close to 21 g C m^−2^ yr^−1^ were reported in Scotland [*Anderson*, 2002] and Canada [*Roulet et al.*, 2007]. Accumulation rates in boreal peatlands are generally lower than in temperate and tropical peatlands. For example, accumulation rates in boreal peatlands in Canada range between 6 and 22 g C m^−2^ yr^−1^ [*Robinson and Moore*, 1999; *Turunen and Turunen*, 2003; *Sannel and Kuhry*, 2009], while accumulation rates in Finland were between 15 and 35 g C m^−2^ yr^−1^ [*Turunen et al.*, 2002; *Ukonmaanaho et al.*, 2006]. In summary, C accumulation rates are, with a few exceptions, greatest in the tropics and decrease with latitude.

The high long-term C accumulation in tropical peatlands may be driven by their high mean NPP, with aboveground biomass production of 1000–1300 g C m^−2^ yr^−1^ [*Nebel et al.*, 2001] and NPP of 1100 g C m^−2^ yr^−1^ [*Chimner and Ewel*, 2005]. Our calculations of NPP_total_ (Table2) and existing data from *Nebel et al.* [2001] and *Chimner and Ewel* [2005] suggest that C inputs from NPP are generally high in tropical wetlands, although there is considerable variability among wetland types. Maximum values for NPP based on litterfall data were 1929 g C m^−2^ yr^−1^ in a forested wetland in Puerto Rico [*Frangi and Lugo*, 1985], while the lowest recorded value was 430 g C m^−2^ yr^−1^ in a floodplain forest in Australia [*Payntner*, 2005]. NPP_total_ was significantly greater in tropical wetlands on organic soils (mean ± SE: 1206 ± 93 g C m^−2^ yr^−1^) than on mineral soils (mean ± SE: 880 ± 77 g C m^−2^ yr^−1^) (*F*_1,49_ = 7.15; *P* = 0.01; Table2). These high rates of productivity generally yield large C stocks, but pool sizes are poorly quantified (Table3).

**Table 3:** Fluxes and Pools of C in Tropical Wetlands on Organic Peat Soil and Mineral Soils; Values are Mean (Standard Deviation; *n*), n/d Refers to No Data, References in Addition to Those in Table1 as Listed Below^a^

	Organic		Mineral	
*Fluxes (g C m^−2^ yr^−1^)*				
Reproductive litter	71.7	(62.6; 17)	73.6	(44.8; 10)
Leaves	333.3	(95.7; 17)	281.2	(86.1; 17)
Fine woody litter	104.9	(51.2; 16)	90.5	(34.1; 9)
Coarse wood	155.0	(183.8; 2)	n/d	
Live wood increment	379.8	(71.7; 2)	547.9	(323.4; 6)
Other litter	28.6	(14.0; 12)	29.0	(2.0; 2)
Fine root production	112.1	(140.3; 7)	n/d	
CO_2_ efflux	−875.1	(481.3; 17)	−901.4	(728.0;18)
CH_4_ efflux	−40.1	(66.1; 15)	−54.0	(52.1; 29)
DOC^b^	−75.5	(17; 2)	−120	(n/d; 1)
*Pools (kg C m^−2^)*				
Leaves	n/d		0.6	(n/d; 1)
Wood	12.4	(4.5; 3)	17.1	(8.2; 4)
Forest floor litter	1.2	(0.9; 8)	0.3	(0.1; 3)
Downed logs	0.8	(n/d; 2)	n/d	
Fine roots	1.9	(2.2; 13)	2.4	(1.7; 5)

a Negative values indicate C losses from the ecosystem.

b From *Richey et al.* [2002], *Moore et al.* [2011], and *Moore et al.* [2013].

A further important aspect of C inputs to tropical wetlands is a more rapid root turnover rate (70% yr^−1^) than in equivalent temperate and boreal systems (55 and 45% yr^−1^, respectively) [*Gill and Jackson*, 2000; *Chimner and Ewel*, 2005]. This observation suggests that C inputs from root turnover might contribute significantly to the high C accumulation rates in tropical wetlands, but data for root production are scarce (Table3).

## 4. Carbon Dioxide and Methane Fluxes From Tropical Swamps

Depending on prevailing environmental conditions, primarily the oxygen content and redox potential of the peat, microbial degradation of organic material in wetlands can induce the release of predominantly CO_2_ or simultaneous release of both CO_2_ and CH_4_. Measurements of daily, monthly, and seasonal variation in gas fluxes show that specific wetlands can switch between production of mainly CO_2_ and a greater contribution of CH_4_ [*Hadi et al.*, 2005; *Jauhiainen et al.*, 2005; *Melling et al.*, 2005a, 2005b; *Wright et al.*, 2013]. Only a few studies have addressed temporal variability in gas fluxes in tropical peatlands, although strong seasonal variation in CH_4_ fluxes has been reported in floodplain wetlands in the Amazon [e.g., *Devol et al.*, 1988; *Bartlett et al.*, 1990]. Gas fluxes can also vary strongly among vegetation types, which in turn are linked to nutrient status [*Wright et al.*, 2013]. Given the diversity of forest types present on tropical wetland soils, this provides a substantial degree of variability. Information on fluxes is almost entirely lacking for many geographical regions; for example, we identified only two papers on CO_2_ emissions and one on CH_4_ emissions from African wetlands. No data were found for gas fluxes from peatlands in the Amazon basin despite their vast spatial extent (150,000 km^2^) [*Lähteenoja et al.*, 2009], although detailed data exist from the floodplains in the region [*Bartlett et al.*, 1988, 1990; *Crill et al.*, 1988; *Devol et al.*, 1988, 1990].

### 4.1. Carbon Dioxide

**Figure 2:**
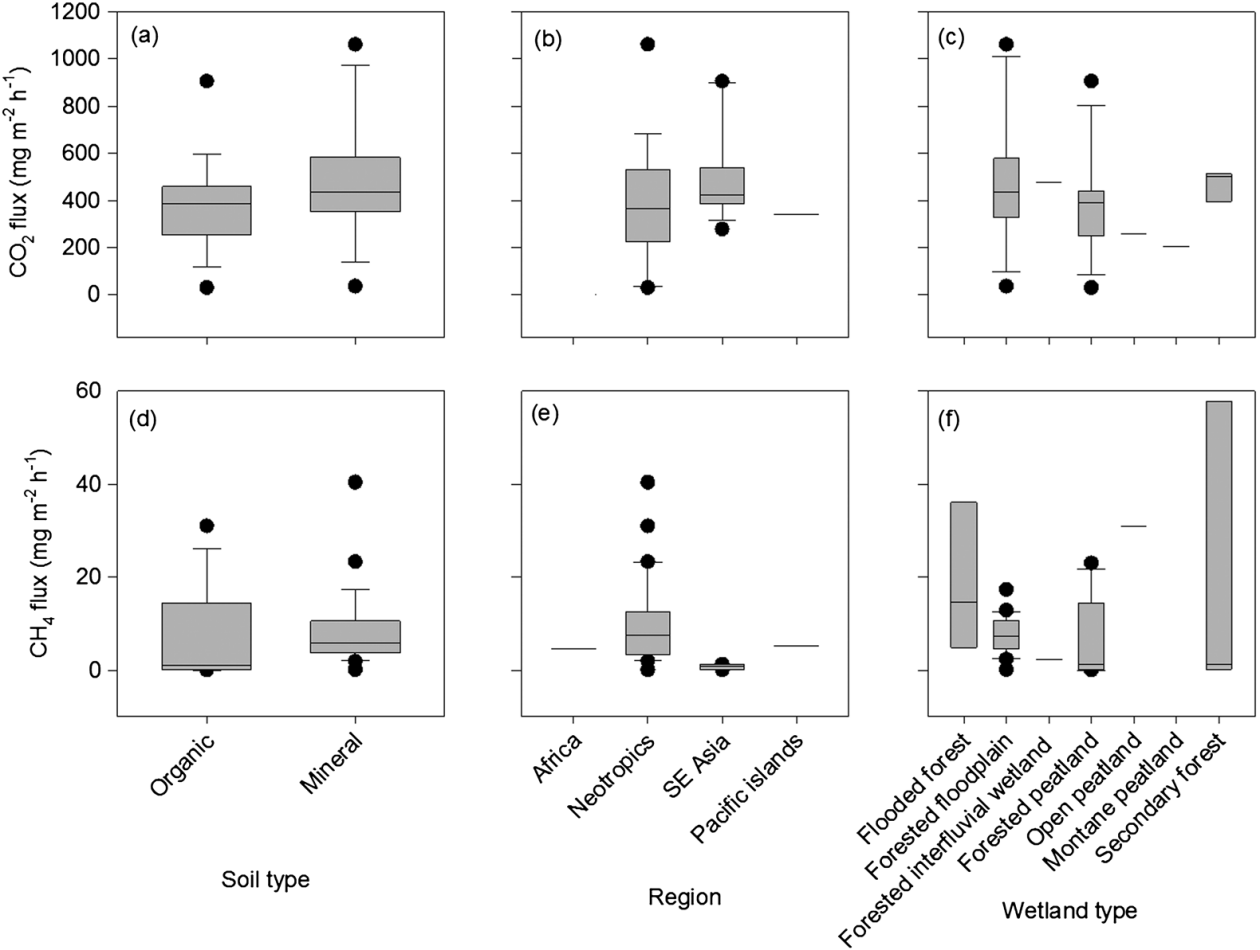
Box plots comparing (a–c) CO_2_ and (d–f) CH_4_ fluxes from different: (Figures2a and 2d) soil types, (Figures2b and 2e) regions, and (Figures2c and 2f) wetland types. The box plots show the lowest and highest observations and the lowest, median, and upper quartiles as well as values which may be considered as outliers. The statistics describing these results are reported in the text.

Fluxes of CO_2_ from forested tropical wetlands vary greatly, with reported values ranging between 30 and 4055 mg m^−2^ h^−1^ (Table4). The lowest values were reported for a palm swamp in Venezuela [*Bracho and San José*, 1990], while values were greatest for a forested peatland in Kalimantan, Indonesia [*Melling et al.*, 2005a]. The majority of available data on CO_2_ fluxes from forested tropical wetlands are from Southeast Asian peatlands, but these tend to be disturbed by human activity, making it difficult to assess regional variation in CO_2_ losses from tropical peatlands. We found no significant differences in CO_2_ efflux among geographical regions (*P* >0.05; Figure2b), although data are absent or very limited for some regions, including both Africa and the Amazon basin, which limits the strength of any conclusions. CO_2_ emission rates tended to be greater in tropical peatlands (Table4) than in temperate and boreal systems [*Silvola et al.*, 1996; *Clair et al.*, 2002; *Bubier et al.*, 2003; *Crow and Wieder*, 2005; *Makiranta et al.*, 2009], although fluxes within specific tropical regions were highly variable and affected by local conditions. Interestingly, the greater range of CO_2_ emissions from flooded forested tropical peatlands [e.g., *Hadi et al.*, 2005; *Melling et al.*, 2005b] were within the same range (i.e., approximately 1000 mg CO_2_ m^−2^ h^−1^) as those found for tropical peatlands with substantially lowered water tables (up to 1 m below the peat surface) [*Couwenberg et al.*, 2010]. Upscaling the CO_2_ fluxes to pantropical wetland areas suggests a release of approximately 4540 ± 1480 Tg CO_2_ year^−1^ (mean ± standard deviation (SD)). This calculation is based on the simplistic assumption that that the CO_2_ flux from mineral soil (Figure2a) is related to the area covered by swamps and floodplains (Table1), and the flux from organic soil (Figure2a) was related to the area covered by peatlands. Substantial additional uncertainty around this mean will arise from current poor understanding of tropical wetland area [*Melton et al.*, 2013; *Lähteenoja et al.*, 2009]. Despite the general accumulation of organic matter in tropical peatlands, there was no significant difference in CO_2_ fluxes between tropical wetlands on organic and mineral soils (*P* >0.05; Figure1a). Furthermore, there was no systematic variation in CO_2_ efflux among wetland types (*P* >0.05; Figure2c).

**Table 4:** Carbon Dioxide (CO_2_) and Methane (CH_4_) Fluxes From Tropical Wetlands Showing the Mean Fluxes^a^ and (Ranges) if Available

Location	Type	Soil Type	CO_2_ Efflux (mg m^−2^ h^−1^)	CH_4_ Efflux (mg m^−2^ h^−1^)	Reference
Kalimantan, Indonesia	Forested peatland	Organic	na	1.1 ± 0.61	*Inubushi et al.* [1998]
Kalimantan, Indonesia	Secondary forest	Organic	501 ± 180 (146–843)	0.18 ± 0.06 (0–1)	*Inubushi et al*. [2003]
Kalimantan, Indonesia	Forested peatland	Organic	317–950	na	*Hirano et al.* [2009]
Kalimantan, Indonesia	Secondary forest	Organic	513	0.19	*Hadi et al.* [2001]
Kalimantan, Indonesia	Secondary forest	Organic	395 (183–4055)	0.50 (0–3.33)	*Hadi et al.* [2005]
Kalimantan, Indonesia	Forested peatland	Organic	399 ± 36 (50–550)	0.16 ± 0.65 (−0.1–0.35)	*Jauhiainen et al.* [2005]
Kalimantan, Indonesia	Forested peatland	Organic	563 (79–1580)	na	*Sundari et al.* (2012)
Sumatra, Indonesia	Forested peatland	Organic	380 ± 55	0.89 ± 0.48	*Furukawa et al*. [2005]
Sumatra, Indonesia	Forested peatland	Organic	278 ± 16	1.21 ± 1.36	*Furukawa et al*. [2005]
Sumatra, Indonesia	Forested peatland	Organic	376 ± 107	0.77 ± 0.27	*Furukawa et al*. [2005]
Malaysia	Forested peatland	Organic	905 (366–1953)	na	*Melling et al*. [2005a]
Malaysia	Forested peatland	Organic	na	0.0029 (−0.006–0.011)	*Melling et al.* [2005b]
Malaysia	Forested peatland	Organic	444		*Murayama and Bakar* [1996]
Thailand	Forest peatland	Organic	na	1.12 ± 2.7 (0.19–12.6)	*Ueda et al.* [2000]
Micronesia	Forested peatland	Organic	396 ± 36 (340–402)	na	*Chimner* [2004]
Mauim, Hawaii	Montane peatland	Organic	285 ± 75		*Chimner* [2004]
Bocas del Toro, Panama	Forested peatland	Organic	212 (11–1694)	23 (−5.35–143)	*Wright et al.* [2011]
Bocas del Toro, Panama	Forested peatland	Organic	238 (62–801)	17 (−3.53–98.3)	*Wright et al.* [2011]
Bocas del Toro, Panama	Open peatland	Organic	259 (7–950)	31 (−6.40–7.88)	*Wright et al.* [2011]
Colon, Panama	Forested peatland	Organic	na	14.4 (0–48)	*Keller* [1990]
Kalimantan, Indonesia	Forested peatland	Organic	na		*Pangala et al.* [2013]
Ka'au, Hawaii	Montane swamp	Organic	127 ± 47	na	*Chimner* [2004]
Orinoco Llanos, Venezuela	Palm swamp	Organic	30 (17–54)	na	*Bracho and San José* [1990]
Sumatra, Indonesia	Forested floodplain	Mineral	410 ± 35	na	*Ali et al.* [2006]
Sumatra, Indonesia	Forested floodplain	Mineral	884 ± 212	na	*Ali et al.* [2006]
Ka'au crater, Hawaii	Forested floodplain	Mineral	na	5.25 ± 0.42 (2.08–14.17)	*Grand and Gaidos* [2010]
La Selva, Costa Rica	Flooded forest	Mineral	na	23.3 ± 14.6	*Nahlik and Mitsch* [2011]
La Selva, Costa Rica	Flooded forest	Mineral	na	40.4 ± 13.1	*Nahlik and Mitsch* [2011]
Earth wetlands, Costa Rica	Secondary forest	Mineral	na	5.7 ± 1.4	*Nahlik and Mitsch* [2011]
Earth wetlands, Costa Rica	Secondary forest	Mineral	na	4.5 ± 0.78	*Nahlik and Mitsch* [2011]
Orinoco, Venezuela	Forested floodplain	Mineral	na	4.6	*Smith et al.* [2000]
Orinoco, Venezuela	Forested floodplain	Mineral	na	10.7 (0–78)	*Smith and Lewis* [1992]
Orinoco, Venezuela	Forested floodplain	Mineral	na	12.8 (0.125–95.3)	*Smith and Lewis* [1992]
Orinoco, Venezuela	Forested floodplain	Mineral	na	7.27 (0–68.7)	*Smith and Lewis* [1992]
Orinoco, Venezuela	Forested floodplain	Mineral	na	10.3 (0–114)	*Smith and Lewis* [1992]
Amazon river, Brazil	Forested floodplain	Mineral	na	4.6 (0.24-31.7)	*Devol et al.* [1988]
Amazon river, Brazil	Forested floodplain	Mineral	na	1.88 (0–8.33)	*Wassmann et al*. [1992]
Amazon river, Brazil	Forested floodplain	Mineral	na	2.29 ± 0.54 (0.014–47.3)	*Devol et al.* [1990]
Amazon river, Brazil	Forested floodplain	Mineral	na	8 ± 1.12	*Bartlett et al.* [1988]
Amazon river, Brazil	Forested floodplain	Mineral	na	5.25 ± 0.83	*Bartlett et al.* [1990]
Amazon river, Brazil	Forested floodplain	Mineral	237	0.1	*Richey et al.* [1988]
Amazon river, Brazil	Forested floodplain	Mineral	36	7.5	*Richey et al.* [1988]
Itu, Negro river, Brazil	Forested interfluvial wetland	Mineral	375	1.9	*Belger et al.* [2011]
Araca, Negro river, Brazil	Forested interfluvial wetland	Mineral	583	2.5	*Belger et al.* [2011]
Pantanal, Brazil	Floodplain	Mineral	na	5.9 ± 13.1 (0.042–91.1)	*Marani and Alvala* [2007]
Pantanal, Brazil	Floodplain	Mineral	554	5.8	*Hamilton et al.* [1995]
Pantanal, Brazil	Floodplain	Mineral	444	2.9	*Hamilton et al.* [1995]
Pantanal, Brazil	Floodplain	Mineral	507	2.9	*Hamilton et al.* [1995]
Pantanal, Brazil	Floodplain	Mineral	317	8.6	*Hamilton et al.* [1995]
Pantanal, Brazil	Floodplain	Mineral	364	8.6	*Hamilton et al.* [1995]
Pantanal, Brazil	Floodplain	Mineral	428	11.5272	*Hamilton et al*. [1995]
Pantanal, Brazil	Floodplain	Mineral	586	11.5	*Hamilton et al.* [1995]
Pantanal, Brazil	Floodplain	Mineral	1062	17.3	*Hamilton et al.* [1995]
Congo river basin, Congo	Flooded forest	Mineral	na	4.41	*Tathy et al.* [1992]

a Error is standard deviation. As the fluxes reported here are from studies extending over different time periods, they should be used for indicative purposes to illustrate the range of fluxes in tropical wetlands. The forested tropical wetlands shown in the table were not managed. Positive fluxes represent a release of CO_2_ or CH_4_ from the peat, and negative CH_4_ fluxes indicate CH_4_ oxidation in the peat. na, not available.

### 4.2. Methane

Estimated fluxes of CH_4_ from peatlands are typically several orders of magnitude lower than those for CO_2_ (Table4). Indeed, CH_4_ emissions are undetectable in some peatlands and uptake from the atmosphere might occur instead. Reported CH_4_ fluxes vary among wetland types (*F*_5,42_ = 6.77, *P* <0.001), ranging from −0.1 to 40 mg CH_4_ m^−2^ h^−1^; the highest values were recorded across a range of wetland systems (Figure2f), including forested peatland and floodplain ecosystems [*Keller*, 1990; *Devol et al*., 1998, 1990; *Nahlik and Mitsch*, 2011; *Wright et al.*, 2011]. CH_4_ fluxes in Southeast Asian forested peatlands were typically lower (<2 mg CH_4_ m^−2^ h^−1^), while the highest, albeit variable, fluxes were reported for the Neotropics (*F*_3,42_ = 12.88; *P* <0.001; Figure2e). For example, fluxes from peatlands in Panama ranged between −5.35 and 143 mg CH_4_ m^−2^ h^−1^ (Table4 [*Wright et al.*, 2011]), highlighting the potential for very high CH_4_ fluxes and marked temporal variability. The highest average CH_4_ emissions were from wetlands on mineral soils (*F*_1,42_ = 6.97, *P* <0.05), with mean fluxes of 8.22 and 6.10 mg CH_4_ m^−2^ h^−1^ in mineral and organic soils, respectively (Figure2d). The high emissions found in tropical wetlands have also been observed in subtropical wetland systems. A maximum emission of 19 mg CH_4_ m^−2^ h^−1^ was found in a subtropical forested floodplain in Australia [*Boon et al.*, 1997], which is comparable to fluxes in swamp forests in the Everglades, USA, [*Bartlett and Harriss*, 1993] and 77 mg CH_4_ m^−2^ h^−1^ from forested floodplains in South Africa [*Otter and Scholes*, 2000]. In contrast, maximum CH_4_ fluxes from flooded temperate and boreal peatlands are lower, ranging between 10 and 14 mg CH_4_ m^−2^ h^−1^ [*Couwenberg et al.*, 2010, and references therein]. Indeed, when comparing the estimated CH_4_ fluxes from tropical wetland to CH_4_ fluxes to higher-latitude wetland (e.g., subarctic and boreal; mean fluxes 4.7 and 3.0 mg CH_4_ m^−2^ h^−1^, respectively) and other types of wetlands (e.g., bog and fens; mean fluxes 4.0 and 3.9 mg CH_4_ m^−2^ h^−1^, respectively), mean tropical CH_4_ fluxes are higher [*Turetsky et al.*, 2014].

Simple upscaling of short-term measurements to the pantropics suggests that approximately 91.6 ± 77 Tg CH_4_ year^−1^ (mean ± SD) is released from tropical wetlands, assuming that the CH_4_ flux from mineral soil (Figure2d) is related to the area covered by swamps and floodplains (Table1), and the flux from organic soil (Figure2d) was related to the area covered by peatlands. Our estimates of CH_4_ emissions from the peat surface of tropical wetlands are within the lower range of fluxes predicted by models [*Melton et al.*, 2013]. In this context, it important to acknowledge the importance of tree stems and canopies for CH_4_ release [*Pangala et al.*, 2013]. This pathway was not included in our calculations, which are therefore likely to underestimate actual fluxes. It will be important to include stem fluxes in future CH_4_ budgets. Additionally, tropical rivers represent an important source of CH_4_ to the atmosphere with recent estimates of CH4 emissions from rivers in the Amazon basin amounting to 0.40 to 0.58 Tg C year^−1^ which should be considered in the context of tropical CH_4_ emissions [*Sawakuchi et al.*, 2014].

The much lower emissions of CH_4_ relative to CO_2_ suggest that only a small component of net C losses result from CH_4_ release. However, given its greater global warming potential compared to CO_2_ [*Meehl et al.*, 2007], CH_4_ emissions at the upper end of the reported emissions range from tropical wetlands are still important from the perspective of radiative forcing.

## 5. Balance Between Carbon Inputs and Outputs

**Figure 3:**
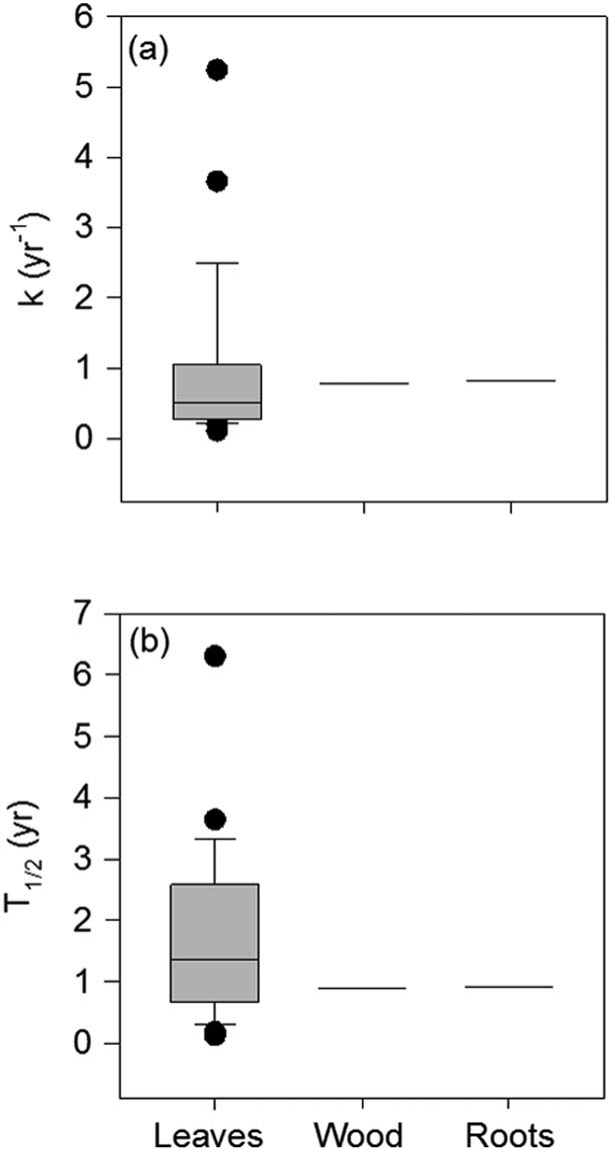
Box plots showing (a) litter decay constants for different tissue types reported in the literature and (b) calculated half times for different tissue types. Data are from in situ decomposition in tropical or subtropical wetlands. Given the small sample size for wood and roots (*n* = 2), only the median values are shown in the graph. The box plots show the lowest and highest observations and the lower, median, and upper quartiles, as well as observations which may be considered as outliers. The statistics describing these results are reported in the text. (Values are from *Furtado et al*. [1980], *Irmler and Furch* [1980], *Frangi and Lugo* [1985], *Brady* [1997], *Rejmankova* [2001], *Del Valle-Arango* [2003], *Gamage and Asaeda* [2005], *Chimner and Ewel* [2005], *Troxler and Childers* [2009], and *Yule and Gomez* [2009]).

The high C effluxes presented above clearly suggest that most of the substantial quantity of C entering wetland systems eventually decomposes and does not contribute to accumulation of C in soil. This is also illustrated by the high litter decay constants (*k*) and short half times (mean 1.6 year) for in situ litter decomposition in tropical and subtropical wetlands (Figure3). Carbon accumulation in tropical wetlands is therefore attributable to the relatively small residual fraction compared to the much larger inputs (litter and root exudates) and outputs (heterotrophic respiration and DOC leaching) of C. This ultimately results in high CO_2_ and CH_4_ emissions from wetlands (Figure2 and Table4), in which environmental conditions are important in determining the proportions released as CO_2_ and CH_4_. For example, drainage of peatland for agriculture result enhances heterotrophic respiration and large CO_2_ losses from SE Asian peatlands (172 Tg C yr^−1^ [*Hooijer et al.*, 2006]) amounting to 12% of C losses arising from deforestation and degradation on the tropics (1.4 Pg year^−1^ [*Houghton*, 2012]). In addition, the compilation of CH_4_ emissions suggests that the low CH_4_ emissions from wetlands in Southeast Asia reported by *Couwenberg et al.* [2010] are not representative of tropical wetlands globally (Figure2 and Table4). It is clear that various natural tropical wetland systems, including peatlands, are potentially significant sources of both CH_4_ and CO_2_ emissions.

Although *k* values for leaf litter decay were high, they differed among tree species and tissue types: the highest and lowest values reported for leaf tissue are, respectively, 5.64 and 0.11 year^−1^ (Figure3). The corresponding values for wood and roots are within the same range as for leaf tissue, but only one study appears to have examined in situ wood and root decay in tropical wetlands [*Chimner and Ewel*, 2005]. Decay constants >1 for some leaf litter types illustrate that some components of the litter input are likely to decompose fully, contributing to the substantial CO_2_ and CH_4_ efflux from tropical wetlands. Based on the existing limited data for different tissue types, it is currently impossible to ascertain whether specific tissue types degrade more slowly than others. However, the low decay constants for leaf litter reported in some studies (Figure3) clearly indicate that leaf materials, as well as wood and roots, contribute to peat formation. As wood and roots were important components for plant biomass production (approximately 50 and 10%, respectively [*Chimner and Ewel*, 2005]), information on their decay rates is needed to establish the relative contribution of tissue types to peat formation.

**Figure 4:**
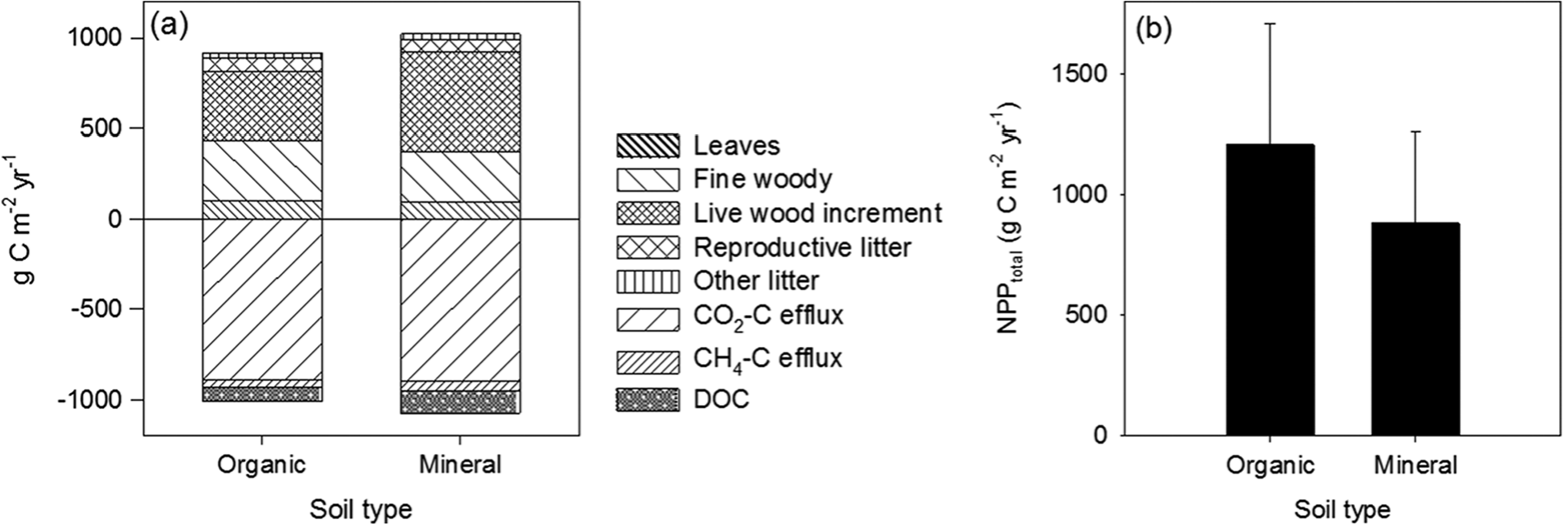
(a) Comparison of mean C inputs and outputs in tropical wetlands on organic and mineral soil, respectively. Note that the number of observations used for the means is highly variable (cf. Table3). There are also some important gaps in the comparison of the C balance between wetlands on organic and mineral soils, namely root (fine and coarse) production and coarse woody litter fall due to lack of data: (b) estimated C inputs (NPP_total_) from fine litterfall data sets (data in Table3) separated between wetlands on organic and mineral soil.

Based on the compilation of litter production and C loss data (Tables2 and 3), C balances were constructed for two types of tropical wetlands: those that are peat-forming, and those occurring on mineral soils (Figure4). Carbon inputs estimated as NPP_total_ (Table2) and from the different litter fractions (Table3) provided comparable results for organic soils (1206 and 1185 g C m^−2^ yr^−1^ for NPP_total_ and NPP_combined_, respectively). As the data set for NPP_total_ was based on a larger number of studies, we used this to calculate NEP. Mean C losses from the soil in the form of respiration (autotrophic and heterotrophic losses and CO_2_ and CH_4_ fluxes combined) and DOC losses for organic soils were lower for organic soils (991 g C m^−2^ yr^−1^) than for mineral soils (1075 g C m^−2^ yr^−1^). In contrast, NPP_total_ was greater in wetlands with organic soils (1206 g C m^−2^ yr^−1^) than for those with mineral soils (880 g C m^−2^ yr^−1^). This resulted in NEP of 215 and −195 g C m^−2^ yr^−1^ for organic and mineral soils, respectively. The estimated NEP is within the range of the long-term C accumulation in tropical peatlands, which ranged between 30 and 300 g C m^−2^ yr^−1^ (see above), but is lower than reported by *Hirano et al.* [2007], who recorded NEP values of 310, 380, and 600 g C m^−2^ yr^−1^ in three consecutive years in a drained peat swamp forest and a papyrus swamp in Uganda (approximately 1000 g C m^−2^ yr^−1^ [*Saunders et al.*, 2012]).

These balances suggest that positive NEP values are reflected by peat accumulation. However, the negative NEP for wetlands on mineral soils clearly indicates that the data must be used with caution; indeed, reliable estimates of NEP cannot be calculated from actual litter production due to the severe limitations in the available database. More specifically, we found only seven studies of fine root production, all on peat soils, and none containing data on coarse root production; these components of the C cycle are therefore not included in Figure4. This is a major concern, given their potentially large contribution to the overall C budget. Based on *Chimner and Ewel* [2005], fine root production amounted to approximately 11% of total plant production in a tropical peatland forest, while *Malhi et al.* [2011] estimated that coarse root production contributed approximately 7% to total plant production in tropical rainforest on mineral soil. Similarly, very few references report data for woody growth, which might represent a large flux of C in tropical wetlands (Table3). Data from *Chimner and Ewel* [2005] suggest that this might introduce an error of 25–30% in estimates of plant production. Omission of belowground and wood increment data from calculations of C balance may therefore lead to underestimations of C inputs of approximately 40–50%.

Similar problems exist with organic C data for fluvial soils. *Ting-Hsuan et al.* [2012] present data for overall regional trends of C export from tropical rivers suggesting that fluvial C losses from tropical rivers are 8.3 g C m^−2^ yr^−1^ with fluxes being estimated to be 2.2, 11.0, and 20.4 g C m^−2^ yr^−1^ for Africa, America, and Asia, respectively. Estimates of carbon exports of 8.5 g C m^−2^ yr^−1^ from the Amazon were presented by *Richey et al.* [1988]. However, these studies do not isolate the contribution from wetlands. Data from *Moore et al.* [2013], including TOC losses of 63 and 97 g C m^−2^ yr^−1^ from intact and disturbed peat swamp forests, respectively, in Kalimantan, suggest a potentially notable contribution of fluvial C losses from NEP calculations for peatland systems in Southeast Asia (approximately 10% increased C losses compared to the above calculations of gaseous losses and 22% compared with local accumulation rates). However, any available TOC or DOC data are integrated over large areas [*Richey et al.*, 2002; *Moore et al.*, 2011, 2013], in contrast to the measurement of litter production and C gas release. Furthermore, high variability of temporal fluvial C in relation to flood and rain events [e.g., *Bass et al.*, 2011], combined with a low number of high-resolution temporal studies, also contribute to the limitations of aquatic C estimates. Given the limited available data, DOC fluxes appear to be of the same order of magnitude as CH_4_ fluxes about an order of magnitude smaller than CO_2_ losses (Figure4 and Table3). Although variation in the reported DOC flux data was substantial between organic and mineral soils systems (60%), the limitations of the available data mean that it is not possible to test whether this is a systematic difference.

Data availability was better for fine litterfall from the canopy, which was used in to calculate NPP_total_. However, the relationship between NPP_total_ and NPP_canopy_ established for lowland rainforests may not be applicable to forested wetlands and may also differ between ombrotrophic and minerotrophic wetlands. Indeed, covariation between nutrient availability, forest composition, and peat depth/organic chemistry [*Phillips et al.*, 1997; *Sjögersten et al.*, 2010] suggests that nutrient availability may provide a strong control of C cycling in tropical wetlands. Care is therefore needed when interpreting these data.

Bearing in mind the data limitation noted above, NPP_total_ appeared to be greater in tropical peatlands than in systems that were not accumulating peat (*F*_1,48_ = 7.15: *P* = 0.01: Figure4b). Data for litterfall and C effluxes were often not available for the same wetland systems, making it difficult to make valid comparisons of C inputs and outputs. Furthermore, the time frame for soil respiration measurements was highly variable, and there were neither long-term data sets on soil CO_2_ efflux nor diurnal variation with respect to plant-mediated gas transport [*Pangala et al.*, 2013]. As a result, comparison of C inputs, which tend to be estimated on an annual basis, and the temporally discrete point measurements of CO_2_ emissions are unbalanced, which is likely to introduce a large error in the estimated NEP.

To assess the C budget of tropical wetlands fully, there is also an urgent need to separate autotrophic and heterotrophic respiration. Based on studies of an *Acacia* plantation on peat soil, *Jauhiainen et al.* [2012] concluded that up to 80% of the CO_2_ efflux from tropical peatlands might originate from root respiration, while work in well-drained tropical forests suggests that root respiration could account for 25–50% of the total soil CO_2_ efflux [*Nottingham et al.*, 2010].

Comparison of our tentative C budgets for tropical wetlands with tropical forest on well-drained soils [*Malhi et al.*, 2011] shows that NPP_total_ from peat forming wetlands is comparable to lowland rainforest, but that NPP_total_ from wetlands on mineral soils are lower. Decomposition rates in the wetland systems were generally lower (approximately 900 and 1350 g C m^−2^ yr^−1^ for wetlands and lowland forests, respectively). Together with the higher NPP_total_ in wetlands on organic peat soils, this suggests that C accumulation in tropical peatlands is driven by a combination of lower decomposition rates and higher NPP.

## 6. Conclusions

Our metaanalysis suggests that greenhouse gas fluxes from tropical wetlands are high, with CH_4_ emissions being highest from mineral soils, although data quality is variable, with substantial data gaps for some regions (Figure1). NEP was greater in peat-forming wetlands than on mineral soils, but missing data for key components of the C balance again add significant uncertainty to our estimates of NEP.

The high CH_4_ emissions, particularly in the Neotropics, might partially explain the high atmospheric CH_4_ concentrations reported for tropical regions [*Mikaloff Fletcher et al.*, 2004a, 2004b; *Meirink et al.*, 2008]. The growing body of recent data for CO_2_ and CH_4_ fluxes from a range of tropical wetlands should be utilized in global wetland models, setting a challenge for the modeling community. However, our ability to assess the role of tropical wetlands in the global C cycle is limited by severe gaps in current understanding of net C inputs (with very limited data on root inputs and woody growth) and outputs (data are largely lacking on DOC losses and separation of autotrophic and heterotrophic respiration), presenting field researchers with an equally important challenge. Without such data, we cannot assess how these ecosystems influence global climate and how their role in the global C cycle may be impacted by future change in land use and climate [*Melton et al.*, 2013].

## Key Points

- Released from tropical wetlands are 90 Tg CH_4_ year−1 and 3860 Tg CO_2_ year−1
- CH_4_ emissions were greatest from mineral soils
- NEP appears greater in peat-forming wetlands than on mineral soil
